# ChsA, a Class Ⅱ Chitin Synthase, Contributes to Asexual Conidiation, Mycelial Morphology, Cell Wall Integrity, and the Production of Enzymes and Organic Acids in *Aspergillus niger*

**DOI:** 10.3390/jof9080801

**Published:** 2023-07-29

**Authors:** Yunqi Zhu, Tong Liu, Yingsi Wang, Guojun Chen, Xiang Fang, Gang Zhou, Jie Wang

**Affiliations:** 1 Guangdong Provincial Key Laboratory of Nutraceuticals and Functional Foods, College of Food Science, South China Agricultural University, Guangzhou 510642, China; superzyq1230@163.com (Y.Z.); pluto0523@126.com (T.L.); cgj1256090697@163.com (G.C.); fxiang@scau.edu.cn (X.F.); 2 Guangdong Provincial Key Laboratory of Microbial Culture Collection and Application, State Key Laboratory of Applied Microbiology Southern China, Institute of Microbiology, Guangdong Academy of Sciences, Guangzhou 510070, China; wongvincy@163.com

**Keywords:** *Aspergillus niger*, chitin synthase, cell wall integrity, conidiation, fungal metabolism

## Abstract

Chitin synthases (CHSs) are vital enzymes for the synthesis of chitin and play important and differential roles in fungal development, cell wall integrity, environmental adaptation, virulence, and metabolism in fungi. However, except for ChsC, a class III CHS, little is known about the functions of CHSs in *Aspergillus niger*, an important fungus that is widely applied in the fermentation industry and food processing, as well as a spoilage fungus of food and a human pathogen. This study showed the important functions of ChsA, a class II CHS, in *A. niger* using multi-phenotypic and transcriptional analyses under various conditions. The deletion of *chsA* led to severe defects in conidiation on different media and resulted in the formation of smaller and less compact pellets with less septa in hyphal cells during submerged fermentation. Compared with the WT, the Δ*chsA* mutants exhibited less chitin content, reduced growth under the stresses of cell wall-disturbing and oxidative agents, more released protoplasts, a thicker conidial wall, decreased production of amylases, pectinases, cellulases, and malic acid, and increased citric acid production. However, Δ*chsA* mutants displayed insignificant changes in their sensitivity to osmotic agents and infection ability on apple. These findings concurred with the alteration in the transcript levels and enzymatic activities of some phenotype-related genes. Conclusively, ChsA is important for cell wall integrity and mycelial morphology, and acts as a positive regulator of conidiation, cellular responses to oxidative stresses, and the production of malic acid and some enzymes, but negatively regulates the citric acid production in *A. niger*.

## 1. Introduction

Chitin, the β-1,4-linked polymer of *N*-acetylglucosamine, is a key cell wall component of fungi and is essential for cell wall organization and integrity, which play important roles in the protection for cells [1,2]. The core of the chitin biogenesis machinery is chitin synthases (CHSs), which catalyze the polymerization of the monomer N-acetyl-D-glucosamine to produce single nascent chitin chains that assemble into chitin nanofibers once they have been secreted [2]. In *Saccharomyces cerevisiae*, three CHS-encoding genes designated as *Chs1*, *Chs2*, and *Chs3* have been identified which play roles in the repairment of damaged chitin during cell separation, primary septum formation, and all other chitin syntheses, respectively [3]. Moreover, cells lacking *chs1* grew normally and showed insignificant changes in the cell wall chitin content, but the disruption of *chs2* resulted in severe growth defects and morphological abnormalities [3]. In filamentous fungi, there are usually approximately 7 CHSs and there can even be more than 20 CHSs, and these can be phylogenetically divided into 7 classes (from class I to VII) [1,4]. In *Neurospora crassa*, *Magnaporthe oryzae*, and *Metarhizium robertsii*, there is one gene for each of the 7 CHS classes, and the disruption of *ChsV*, *ChsVI*, or *ChsVII* in *N. crassa*, *ChsI*, *ChsII*, *ChsV*, or *ChsVII* in *M. robertsii*, and *ChsII* or *ChsVII* in *M. oryzae* has been shown to cause growth defects under heat stress [1]. Moreover, only ChsV and ChsVII contributed to fungal virulence, morphogenesis, and conidiation in *M. robertsii* [1]. Additionally, the inactivation of *Chs1*, a class III chitin synthase, led to slow growth, aberrant hyphal morphology, and a decrease in chitin synthase activity in *N. crassa* [5]. In *Metarhizium acridum*, seven chitin synthases (ChsI-VII) were identified. The gene knockouts of *ChsV* and *ChsVII* resulted in an aberrant distribution of chitin, the deletion of *ChsI*, *ChsIII*, and *ChsIV* caused delayed conidial germination, and disrupted mutants of *ChsII* and *ChsV* germinated more rapidly, but all CHS-encoding genes positively impacted the conidial yield. Moreover, ChsIII, ChsV, and ChsVII were required for cell wall integrity and fungal virulence, and ChsIII and ChsVII also contributed to sensitivities to heat and UV-B stress [6]. In *Aspergillus fumigatus*, eight CHS-encoding genes were found and designated as *ChsA* (Class I), *ChsB* (Class II), *ChsC* and *ChsG* (Class III), *CsmA* (Class V), *CsmB* (Class VI), *ChsF* (Class IV), or *ChsD* (Class VII) [7]. ChsA played positive roles in conidiation, chitin content, and tolerance to high temperature, but negative roles in the tolerance to Congo red (a cell wall-disturbing agent) [7]. ChsB was positively associated with conidiation, chitin content, and tolerance to Congo red [7]. ChsC is positively related to conidiation, chitin content, and tolerance to high temperature but negatively related to tolerance to Congo red [7]. ChsG was important for fungal growth, hyphal and conidial morphology, tolerance to high temperature, CHS activity, and chitin content, but negatively regulated tolerance to Congo red, Calcofluor white (a cell wall-disturbing agent), and Nikkomycin Z (an antifungal drug) [7]. Δ*csmA* displayed reduced growth under normal conditions and high temperatures; defects in conidiation, conidial morphology, CHS activity, conidial chitin, and virulence; and an increase in sensitivity to Congo red and Calcofluor white, as well as tolerance to Nikkomycin Z [7]. Δ*csmB* exhibited defects in conidiation, CHS activity, conidial chitin, and virulence, and an increase in sensitivity to high temperature, Congo red, and Calcofluor white, as well as tolerance to Nikkomycin Z [7]. Δ*chsD* exhibited an increase in mycelial chitin, and Δ*chsF* exhibited a decrease in conidial chitin [7]. In *Aspergillus nidulans*, there were eight CHS-encoding genes. Among them, the deletion of *ChsC* (a class I CHS-encoding gene) or *ChsD* (a class IV CHS-encoding gene) caused no significant change in phenotype, the deletion of *ChsA* (a class II CHS-encoding gene) induced a slight reduction in conidiation efficiency, the deletion of *ChsB* (a class III CHS-encoding gene) resulted in extremely small colonies with highly branched hyphae, and the deletion of *CsmA* (a class V CHS-encoding gene) or *CsmB* (a class VI CHS-encoding gene) led to the formation of swollen hyphae and intrahyphal hyphae [8]. In *Fusarium graminearum*, nine CHS-encoding genes were found, and it was reported that Chs1 (Class I), Chs5 (class V), and Chs7 (class VII) negatively affected fungal growth, development, and pathogenicity, and Chs8 (class VIII) positively affected the accumulation of chitin, chitin synthase activity, the tolerance to sodium dodecyl sulfate and salicylic acid, deoxynivalenol production, and pathogenicity [9]. In *Penicillium digitatum*, seven CHSs (from Chs I to VII) were identified, and the disruption of *ChsVII* caused defects in fungal growth, conidia production, and morphology, an increase in chitin content and susceptibility to Calcofluor white, sodium dodecyl sulfate, hydroxide peroxide, and commercial fungicides, and a reduction in virulence [10]. In *Penicillium chrysogenum*, the inactivation of *chs4*, a Class III CHS-encoding gene, resulted in a slow growth rate, shorter and more branched hyphae, reduced conidiation, and increased penicillin production [11]. These findings revealed that chitin synthases play important roles in cellular growth and development, cell wall integrity, environmental adaptation, metabolism, and pathogenicity in fungi, but the roles differed from each CHS-encoding gene, whose functions were different in different fungi.

*Aspergillus niger* is an important filamentous ascomycete fungus that is widely applied in the fermentation industry and food processing to produce various primary and secondary metabolites, such as organic acids, bioactive compounds, and enzymes; to improve the sensory characteristics of fermented foods and bioavailability of polyphenols; and to detoxify some mycotoxins such as aflatoxin B1, which have great economic and research significance [12,13,14,15]. Additionally, *A. niger* could cause rot in vegetables and fruits, which leads to a huge waste of resources [16]. More seriously, *A. niger* might cause pulmonary aspergillosis, which is harmful to human health [17]. Therefore, in order to improve the fermentation performance of, and reduce contamination or infection by *A. niger*, it is necessary to unveil its molecular mechanisms which are involved in the regulation of fungal growth and development, metabolism, and pathogenicity. There exist eight CHS-encoding genes in the genome database of *A. niger* [15], and the silencing of *chsC*, a class III CHS-encoding gene, resulted in a reduction in conidiation efficiency and the chitin content of the cell wall, as well as increases in the compactness of the mycelial pellets and the production of citric acid [14], confirming the indispensability of the chitin synthase in conidiation, mycelial morphology, and the production of citric acid in *A. niger*. These results suggest that other CHS-encoding genes might play vital roles in fungal development and metabolism of *A. niger*. Moreover, previous studies have shown that *chsA* (a class II CHS-encoding gene) and *chsC* might be the explanation for the formation of dispersed mycelia rather than the pellets caused by epigallocatechin-3-gallate (EGCG) in *A. niger* RAF106, which might also result in changes in the production of some metabolites such as cellulases and pectinases [18]. However, little is known about the roles of *chsA* in *A. niger*. Therefore, this study aimed to elucidate the functions of *A. niger chsA* through analysis of the changes in multi-phenotypes, the production of various enzymes and organic acids, and the transcriptional expression of phenotype-related genes between the wild-type strain and *chsA* deletion mutants. The results demonstrated that the *chsA* gene was closely associated with asexual conidiation, mycelial morphology, cell wall integrity, cellular response to oxidative stresses, and the production of some enzymes and organic acids, which will be helpful in enriching the understanding of the Class II CHSs, improving their fermentation performance, and controling the contamination of *A. niger*.

## 2. Materials and Methods

### 2.1. Microbial Strains and Culture Conditions

The wild-type strain (WT) *A. niger* RAF106 (CGMCC No. 9608) was cultivated in potato dextrose agar (PDA; 20% potato (infusion form), 2% glucose, and 1.5% agar), Sabouraud dextrose agar (SDAY; 4% dextrose, 1% peptone, 1% yeast extract, and 1.5% agar), and Czapek agar (CZA; 3% sucrose, 0.3% NaNO_3_, 0.1% K_2_HPO_4_, 0.05% KCl, 0.05% MgSO_4_, 0.001% FeSO_4_, and 1.5% agar) at 30 °C for fungal growth and conidiation. PDA was used for phenotypic assays. *Escherichia coli* DH5a from Invitrogen (Shanghai, China) grown in Luria-Bertani medium at 37 °C was used to propagate the plasmids. *Agrobacterium tumefaciens* AGL-1 cultivated at 28 °C in YEB medium [19] was used as a T-DNA donor for fungal transformation.

### 2.2. Cloning and Analysis of chsA in A. niger

The sequence of *chsA* (gene08422) was amplified from the WT using paired primers (Table S1) and was sequenced at Invitrogen. The deduced protein sequence was structurally analyzed via an online BLAST available online: http://blast.ncbi.nlm.nih.gov/blast.cgi (accessed on 17 June 2023), and phylogenetically analyzed with class II chitin synthases from other fungi using a neighbor-joining method in MEGA 7.0 software (Mega Limited, Auckland, New Zealand). The molecular size and theoretical isoelectric point of ChsA were predicted using the available online: https://web.expasy.org/protparam/ (accessed on 20 June 2023) and the subcellular localization was predicted using the available online: https://wolfpsort.hgc.jp/ (accessed on 20 June 2023).

### 2.3. Construction of chsA Mutants

*ChsA* was deleted by homologous replacement [20]. Briefly, the expression cassette, including the promoter and open reading frame of *hygB* (conferring resistance to hygromycin B), was amplified from pSilent-1 with paired primers (Table S1) and cloned into the p0380-bar cut with *Sac*I/*Xho*I, forming p0380-*hygB*. The *chsA* disruption vector was constructed by cloning the 5′ and 3′ fragments of the gene amplified from the WT genome via PCR with paired primers (Table S1) into p0380-*hygB* cut with *Xma*I/*Sma*I and *Xho*I/*Xba*I, respectively. The disruption plasmid was introduced into WT via *Agrobacterium*-mediated transformation [19]. Putative mutant colonies obtained from a selective medium with cefotaxime (300 μg/mL) and hygromycin B (50 μg/mL) were identified via PCR and quantitative real-time PCR (qRT-PCR) with paired primers (Table S1).

### 2.4. Determination of Fungal Growth and Conidiation

A suspension of 1 μL of 1 × 10^6^ conidia/mL was spotted on plates (9 cm diameter) of SDAY, CZA, and PDA media. After a 2-day incubation at 30 °C, the diameter of each colony was cross-measured as an index of fungal growth. To assess the capacity for conidiation, a suspension of 100 μL of 1 × 10^6^ conidia/mL was spread evenly on SDAY, CZA, and PDA plates and incubated at 30 °C. From day 3, colony discs (5 mm diameter) were cut randomly from the plates with a cork borer and individually immersed in 1 mL of 0.02% Tween 80 solution by supersonic vibration for 10 min. The suspension was filtered through four layers of lens tissues to remove hyphal debris, and the conidial concentration was measured using a hemocytometer and then converted to the number of conidia per cm^2^ of the colony. Meanwhile, 3-day cultures were cut using a razor blade and observed under a microscope to assess the morphological changes in conidiophores.

### 2.5. Assays for Pellet Formation and Condidial Agglomeration

A suspension of 1 mL of 1 × 10^8^ conidia/mL was added into 100 mL of potato dextrose broth (PDB; agar-free PDA) and shaken at 180 rpm for 48 h at 30 °C. During the first 4 h incubation, conidial agglomeration was observed under a microscope at 2 h intervals. Morphological changes in fungal mycelia were examined under a microscope at 12 h intervals.

### 2.6. Assessments for Cellular Responses to Chemical Stresses

A suspension of 1 μL of 10^6^ spores/mL was spotted on plates (9 cm diameter) of PDA alone (control) and PDA media supplemented with different concentrations of Congo red (CR; 2 mg/mL and 3 mg/mL), sodium dodecyl sulfate (SDS; 0.03%, and 0.04%), NaCl (1.2 M), KCl (1.2 M), menadione (MND; 1 μM and 2 μM), and H_2_O_2_ (6 mM and 12 mM), respectively. After a 2-day incubation at 30 °C, the diameter of each colony was measured as mentioned and the relative growth inhibition was calculated as (D_c_ − D_t_)/D_c_ × 100%, where D_c_ and D_t_ are colony diameters on the control plates and tested plates with the responding chemical stress for each strain, respectively.

### 2.7. Examination of Cell Wall Changes

Cell wall isolation and chitin content analysis were examined as described previously [21]. Briefly, a suspension of 1 mL of 10^8^ spores/mL was added into 100 mL of complete liquid medium (CM; 1% glucose, 0.1% tyrosine, and 0.5% yeast extract) and shaken at 180 rpm at 30 °C. After a 17 h incubation, mycelia were harvested, ground in liquid nitrogen, and washed with 50 mL of 1 M NaCl and 50 mL of high-quality deionized water (MQ) to remove intracellular debris and proteins. The cell wall suspension was centrifuged at 5000 rpm for 10 min to obtain the cell wall. Chitin in the cell wall was measured as total glucosamine based on the principle of the Morgan-Elson protocol [22].

The fragility of the cell wall was assayed as described previously with slight modification [23]. Briefly, a suspension of 1 mL of 10^8^ spores/mL was added into 100 mL of Sabouraud dextrose broth (SDB; agar-free SDAY) and shaken at 180 rpm at 30 °C. After a 17 h incubation, mycelia (100 mg) were harvested, washed twice with 0.1 M phosphate buffer (PBS; pH 6.8), resuspended in 2 mL of 0.8 M KCl containing 0.1% snailase (Beyotime, Shanghai, China) and 1.5% lysing enzyme (Beyotime, Shanghai), and incubated at 37 °C for 3.5 h with shaking at 150 rpm. The concentration of protoplasts released from the hyphal cells was measured using a hemocytometer plate and considered as an index of cell wall fragility.

The cell wall of aerial conidia was observed under transmission electron microscopy (TEM) as described previously [24]. Briefly, conidia were harvested from 5-day cultures cultivated on the PDA plates, washed with PBS, fixed in the 2.5% glutaraldehyde buffer, washed with PBS, dehydrated in a gradient ethanol series, embedded in Spurr resin, dyed with uranyl acetate solution and a lead solution supplemented with citric acid, and observed under a Talos L120C TEM (FEI).

### 2.8. Calcofluor White Staining

Calcofluor white (CFW) staining of the hyphae was performed as described previously [25]. Briefly, a suspension of 1 mL of 10^8^ spores/mL was added into 100 mL PDB with 50 glass beads and shaken at 180 rpm at 30 °C. After a 24 h incubation, the hyphae were collected and placed on the cover slide containing a 20 μL droplet of 10 μg/mL CFW. After a 5 min incubation, the CFW fluorescence was observed under a fluorescence microscope (Axio Observer A1, Carl Zeiss, Oberkochen, Germany).

### 2.9. Quantitative Analysis of Antioxidant Enzymes

Aliquots of 100 mg hyphal mass from 3-day-old PDA cultures grown at 30 °C were ground in liquid nitrogen, re-suspended in PBS buffer, and centrifuged at 4 °C. The supernatant was collected and served as the crude enzymes. The activities of superoxide dismutases (SODs) and catalases (CATs) were measured by total superoxide dismutase assay kit with WST-8 (S0101S) and catalase assay kit (S0051), respectively, and the protein concentration was measured by BCA Protein Assay Kit (Beyotime, Shanghai, China) according to the manufacturer’s directions. The number of SODs and CATs was measured at 450 and 520 nm, respectively, under a microplate reader (Molecular Devices VersaMax, San Jose, CA, USA). One unit of SOD activity was defined as the SOD amount required to inhibit 50% of the coupling reaction of xanthine. One unit of CAT activity was defined as 1 μmol H_2_O_2_ consumed per minute at 25 °C and pH 7.0.

### 2.10. Assays for Amylases, Cellulases, Pectinases and Proteases

The production of amylases, cellulases, and pectinases was measured as described previously [18]. Briefly, a suspension of 1 mL of 10^8^ spores/mL was added into 100 mL Wheat Bran straw medium (WBS; 55 g/L rice straw flour, 1.6 g/L (NH_4_)_2_SO_4_, 0.2 g/L MgSO_4_, 30 g/L peptone, 2 g/L KH_2_PO_4_, 0.01 g/L CaCl_2_, 0.002 g/L ZnCl_2_, 0.2 g/L urea, pH 5.0). After 3-day incubation with shaking at 30 °C, the supernatant was collected and served as the crude enzymes. For cellulase activity, 0.05 mL of crude enzymes were incubated with 0.95 mL of 1% sodium salt carboxy methyl-cellulose (CMC-Na) solution in 0.05 M sodium citrate buffer at 50 °C for 20 min. For amylase activity, 0.05 mL of crude enzymes were incubated with 0.45 mL of 1% starch solution in 0.05 M sodium citrate buffer at 50 °C for 30 min. For pectinase activity, 0.05 mL of crude enzymes were incubated with 0.45 mL of 0.5% pectin solution in 0.05 M sodium citrate buffer at 50 °C for 30 min. The reactions were stopped by adding 0.75 mL of 3,5-dinitro salicylic acid (DNS) reagent and immersing the reaction tube in boiling water (100 °C) for 10 min. One unit of enzymatic activity was defined as the amount of enzyme required to produce 1 mmol of D-glucose for cellulase activity, 1 mg of D-glucose for amylase activity, and 1 mg of D-galacturonic acid for pectinase activity by hydrolyzing the raw substrate per minute under one of the specified assay conditions.

The protease production was measured as described previously [26]. Briefly, the supernatant was collected from 3-day cultures of *A. niger* incubated in WBS and served as the crude enzymes. Then, 0.5 mL of crude enzymes were incubated at 40 °C for 5 min and mixed with 1 mL of 2% casein which was incubated at 40 °C for a while. After a 10 min incubation at 40 °C, the reaction was stopped by adding 2 mL of 0.4 M trichloroacetic acid solution. After a 15 min incubation, the supernatant was collected, supplemented with 5 mL of 0.4 M sodium carbonate and 1 mL of 2 M Folin-phenol solution, and incubated at 40 °C for 20 min. The absorbance at 680 nm was measured and one unit of enzymatic activity was defined as the amount of enzyme required to produce 1 μg of tyrosine by hydrolyzing the raw substrate per minute under one of the specified assay conditions.

### 2.11. HPLC Analysis of Citric and Malic Acid Production

The production of citric and malic acid was analyzed using HPLC as described previously [27]. Briefly, a suspension of 1 mL of 10^8^ spores/mL was added into 100 mL WBS. After 3-day incubation with shaking at 30 °C, the supernatant was collected and filtered through membrane filters (0.22 μm). Aliquots of 10 μL of filtered supernatant were injected into an HPLC system equipped with a YMC C18 column (150 mm × 4.6 mm, 5 μm) and a UV detector set at 280 nm and eluted with the mobile solvent consisting of 10% methanol at a constant flow rate of 0.5 mL/min at 30 °C. The concentration of citric and malic acid was determined using external calibration curves of the standard solution at different concentrations.

### 2.12. qRT-PCR

Cultures for transcriptional expression of conidiation-related genes were initiated by spreading aliquots of 1 mL of 10^6^ spores/mL on cellophane-attached PDA plates. After a 1-day incubation at 30 °C, mycelia were harvested, ground in liquid nitrogen, and used to extract total RNA under the action of a TRIzol^®^ Reagent (Invitrogen, Carlsbad, CA, USA). Total RNA was transcribed reversely into cDNA with a PrimeScript RT reagent kit (TaKaRa, Kyoto, Japan). Tenfold dilutions of each cDNA were used as templates to analyze the expression of tested genes via qRT-PCR with paired primers (Table S2) using SYBR^®^ Premix Ex Taq™ (TaKaRa, Kyoto, Japan). *β*-*tubulin* was used as an internal standard. The relative transcription level of each gene was defined as the ratio of its transcript in each mutant over that in WT using the 2^−ΔΔCt^ method [28].

### 2.13. Assay for the Infection Ability of A. niger on Apple

Healthy apples (175 g/apple) with uniform size, color, and weight were washed with tip water, and the surface was sterilized with 75% ethanol. Four wounds (2 mm in diameter and 3 mm deep) were made on the surface of each apple with a sterile yellow tip. After air-drying, aliquots of 5 μL of conidial suspension (final concentration of 10^6^ spores/mL) were injected into each wound. The apples were placed in sealed containers and incubated continuously at 25 °C. The wound lesion was observed and photographed every day.

### 2.14. Statistical Analysis

All the experiments were conducted in triplicate. Results from replicates were expressed as mean ± standard deviation (SD) and the data analysis was subjected to a one-factor analysis of variance, followed by Tukey’s HSD test. *p* < 0.05 was considered a significant difference in all experiments.

## 3. Results

### 3.1. Bioinformatic Characteristics of ChsA in A. niger

**Figure 1:**
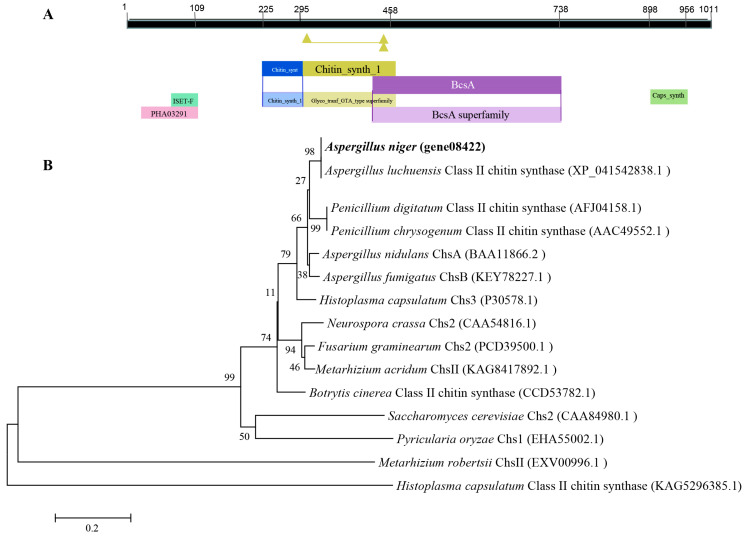
Structural features and phylogenetic analysis of ChsA in *A. niger*. (**A**) The conserved domains of *ChsA*. (**B**) Phylogenetic tree constructed for ChsA in *A. niger* and other fungal Class II CHSs in the NCBI database (accession codes given in parentheses) using a neighbor-joining method. Scale bar: branch length proportional to genetic distance.

A single ChsA sequence (Gene ID: gene08422) was found in the genome database of *A. niger* RAF106. Three introns consisting of 56, 60, and 51 bases, respectively, were found by comparing the genomic DNA and cDNA sequences. The open reading frame of *chsA* consists of 3036 bases, which encode 1011 amino acids with a molecular weight of approximately 112.48 kDa and an isoelectric point of 8.01. ChsA was predicted to locate in the plasma membrane and featured typical domains of CHS family members named Chitin_synth_1 (pfam01644) and Chitin_synth_C (cd04190), as well as three other conserved domains including the BcsA superfamily, which is the catalytic subunit of cellulose synthase and poly-beta-1,6-N acetylglucosamine synthase, Caps_synth_CapC superfamily, which is essential for the production of gamma-polyglutamic acid, and PHA03291 superfamily, which envelopes glucoprotein I (Figure 1A). Comparisons of the amimo acid sequences with other 24 CHSs belonging to the seven classes (from class I to VII) from *A. fumigatus*, *A. nidulans*, and *Aspergillus luchuensis*, as well as phylogenetic analysis, revealed that ChsA was subdivided into the Class II clade (Figure S1A). Moreover, phylogenetic analysis demonstrated that ChsA shares 42.07–99.59% of its sequence identity with 15 other class II CHSs found in fungi; it shows the highest similarity (99.59%) to a class 2 chitin synthase found in *A. luchuensis*, and the lowest similarity (42.07%) to Chs1, found in in *Pyricularia oryzae* (Figure 1B).

### 3.2. Effects of the Deletion of chsA on Radial Growth, Conidiation, and Mycelial Morphology

The deletion of *chsA* was conducted in the WT by homogeneous replacement via *Agrobacterium*-mediated transformation (Figure S1B). Three expected disruption mutants were confirmed by PCR and qRT-PCR analyses (Figure S1C), which demonstrated that *chsA* is nonessential for fungal viability in *A. niger*.

**Figure 2:**
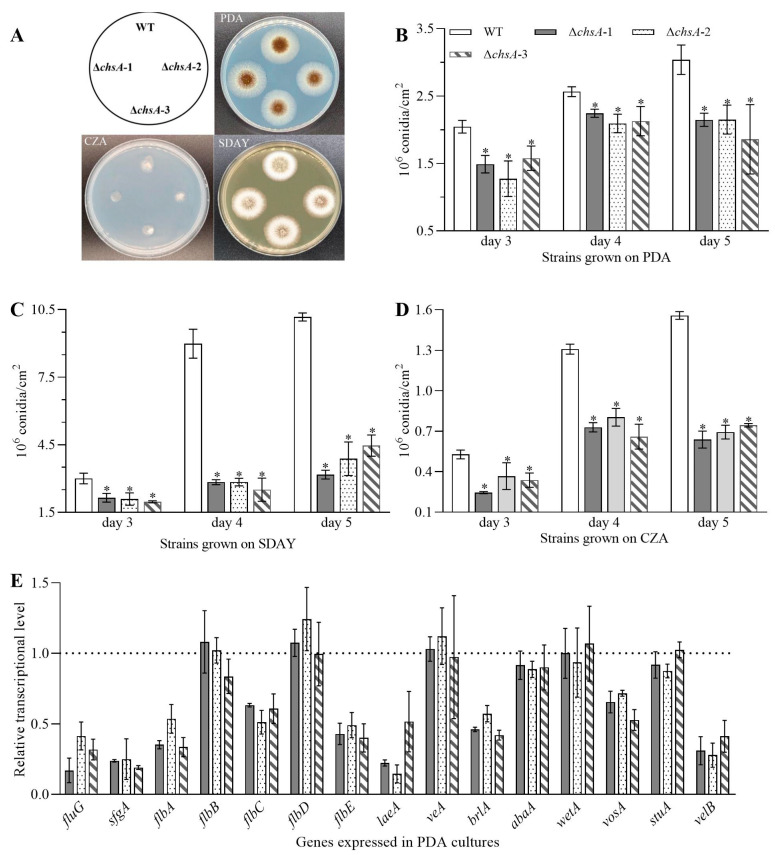
Roles of *chsA* in the asexual development of *A. niger*. (**A**) Images of fungal colonies after two-day growth on PDA, SDAY, and CZA plates at 30 °C. (**B**–**D**) Conidial yields during the period of incubation on PDA, SDAY, and CZA plates at 30 °C, respectively. (**E**) Relative transcript levels of conidiation-associated genes in Δ*chsA* versus WT. All cDNA samples were derived from one-day-old hyphal cells incubated on PDA plates and analyzed via qRT-PCR. The dashed line represents the transcriptional levels of genes in the WT. Asterisked bars in each bar group differ significantly from those unmarked (Tukey’s HSD, *p* < 0.05). Error bars: SDs from three replicates.

After a two-day incubation on PDA, SDAY, and CZA plates, all of the disruption mutants of *chsA* exhibited similar diameters of fungal colonies to the WT (Figure 2A). However, the deletion of *chsA* caused severe defects in the conidiation capacity. Compared with the WT estimates of three-, four-, and five-day-old cultures, the mean yields of conidia in Δ*chsA* were reduced by 22.87–37.75%, 12.55–18.37%, and 29.23–38.85%, respectively, for the PDA cultures (Figure 2B), 28.53–34.43%, 68.38–72.10%, and 56.13–68.79%, respectively, for the SDAY cultures (Figure 2C), and 30.41–53.56%, 38.57–49.58%, and 52.21–59.00%, respectively, for the CZA cultures (Figure 2D). Accompanied by the defects in conidial yields, Δ*chsA* showed sparser and smaller conidiophores than the WT according to the images observed under a microscope of samples obtained from the colonies on day three (Figure S2). Moreover, the transcripts of conidiation-required genes, including *fluG*, *sfgA*, *flbA*, *flbC*, *flbE*, *laeA*, *brlA*, *vosA*, and *velB*, were suppressed by 28.26–85.42% in Δ*chsA* compared with those in the WT when grown on PDA plates (Figure 2E).

**Figure 3:**
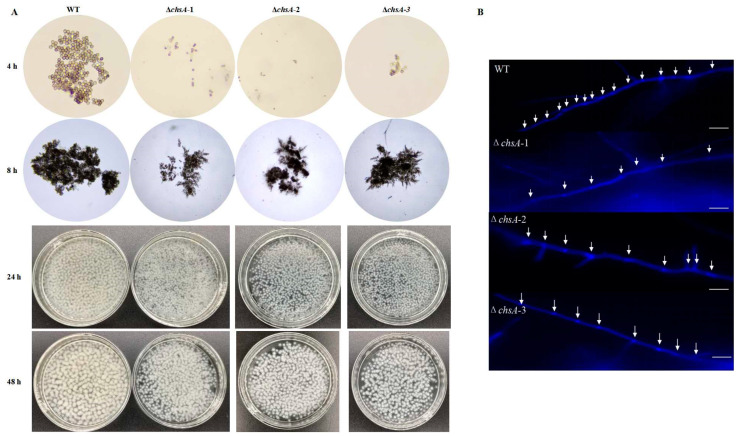
Images of conidial agglomeration (**A**), pellets morphology (**A**), and CFW staining (**B**) (scale: 100 μm) observed under a microscope/fluorescence microscope when the suspension of conidia from the WT and Δ*chsA* were incubated at PDB with shaking at 30 °C for 48 h. The arrows showed the positions of the septa.

Additionally, the images analysis demonstrated that the deletion of *chsA* resulted in the formation of smaller and less compact pellets during submerged fermentation in PDB media, compared with the WT (Figure 3A). Correspondingly, the deletion of *chsA* inhibited conidial agglomeration. For the WT, a larger number of conidia aggregated after a 4 h incubation, and more conidia aggregated, germinated, and clustered in the group after 8 h incubation; however, for the deletion mutants of *chsA*, only a few conidia aggregated after 4 h incubation and the clusters of conidia and hyphae were smaller than that in the WT after 8 h incubation (Figure 3A). Moreover, CFW staining demonstrated that the number of the septa in the hyphae of Δ*chsA* was less than that in the WT (Figure 3B). These findings demonstrated that *chsA* participated in the maintenance of mycelial morphology during submerged fermentation.

### 3.3. Essential Roles of chsA in Cell Wall Integrity

**Figure 4:**
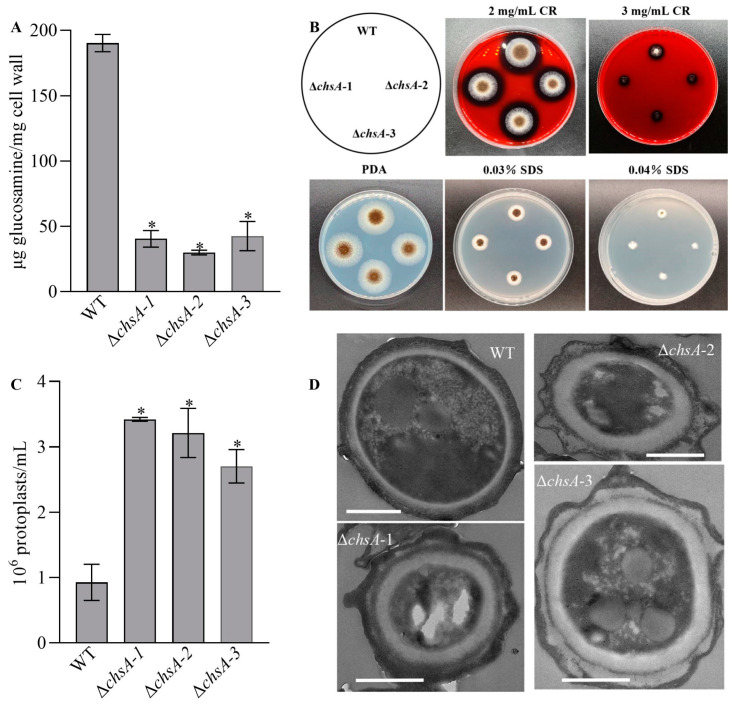
Contributions of *chsA* to cell wall integrity of *A. niger*. (**A**) The content of total glucosamine based on the principle of the Morgan-Elson protocol. (**B**) Images of fungal colonies after two-day growth on PDA and PDA supplemented with Congo red (2 and 3 mg/mL) or SDS (0.03% and 0.04%) at 30 °C. (**C**) The number of protoplasts released from mycelial cells after 3.5 h treatment with cellwall-degrading enzymes. (**D**) TEM images for ultrathin sections of conidia (scales: 1.0 μm). Asterisked bars in each bar group differ significantly from those unmarked (Tukey’s HSD, *p* < 0.05). Error bars: SDs from three replicates.

The deletion of *chsA* led to remarkable cell wall damage. Compared with the WT, the deletion mutants of *chsA* exhibited a 77.61–84.26% decrease in the mean content of *N*-acetylglucosamine (Figure 4A), suggesting that *chsA* positively contributed to the chitin content in *A. niger*. Moreover, Δ*chsA* grew much slower than the WT under the stresses of 2 and 3 mg/mL Congo red and also showed a slight decrease in the diameter of the colony under the stress of 0.04% SDS, compared with that in the WT (Figure 4B). Interestingly, more protoplasts were released from hyphal cells after a 3.5 h incubation with cell lysing enzymes in Δ*chsA* than that in the WT (Figure 4C). Compared with the WT, the number of released protoplasts increased by 1.92–2.69-fold on average in Δ*chsA*, suggesting that the deletion of *chsA* led to an increase in the fragility of the hyphal wall. Furthermore, the TEM images showed that the outermost layer of the conidial wall became looser and thicker in all of the disruption mutants than that in the WT (Figure 4D). These results strongly indicated that *chsA* played important roles in the development of the cell wall in *A. niger*.

### 3.4. Contribution of chsA to Chemincal Stresses and Virulence in A. niger

**Figure 5:**
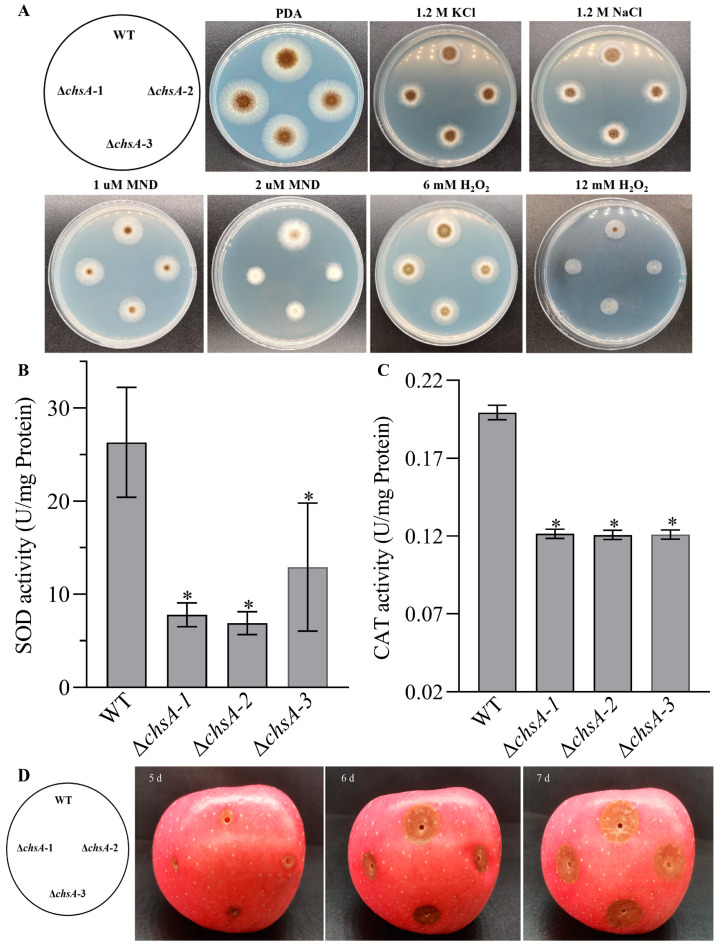
Changes in the adaptive responses to oxidative and osmotic stresses and virulence caused by the deletion of *chsA* in *A. niger*. (**A**) Images of fungal colonies after two-day growth on PDA and PDA supplemented with KCl (1.2 M), NaCl (1.2 M), menadione (MND: 2 and 3 μM), or H_2_O_2_ (6 and 12 mM) at 30 °C. (**B**,**C**) The total activities of SODs (**B**) and CATs (**C**), respectively. (**D**) The infection ability on apple. Asterisked bars in each bar group differ significantly from those unmarked (Tukey’s HSD, *p* < 0.05). Error bars: SDs from three replicates.

The deletion of *chsA* caused increases in sensitivities to the oxidants menadione and H_2_O_2_ during colony growth (Figure 5A). Compared with the estimates in the WT, the percentage of relative growth inhibition increased by 12.85–63.79% under the stresses of 1 and 2 μM menadione, respectively, and 14.34–19.78% under the stresses of 6 and 12 mM H_2_O_2_, respectively. Correspondingly, the SODs and CATs activities were lowered by 51.00–73.82% and 39.07–39.44% in Δ*chsA*, compared with those in the WT (Figure 5B,C). However, Δ*chsA* displayed similar growth to the WT under the stresses of 1.2 M NaCl and KCl and a similar lesion diameter to the WT on the apples (Figure 5A,D). These findings indicate that *chsA* was a positive regulator for cellular tolerance to the oxidants but was not involved in the fungal response to osmotic stresses and fungal pathogenicity in *A. niger*.

### 3.5. Changes in the Production of Amylases, Pectinases, Proteases, Cellulases, and Organic Acids

**Figure 6:**
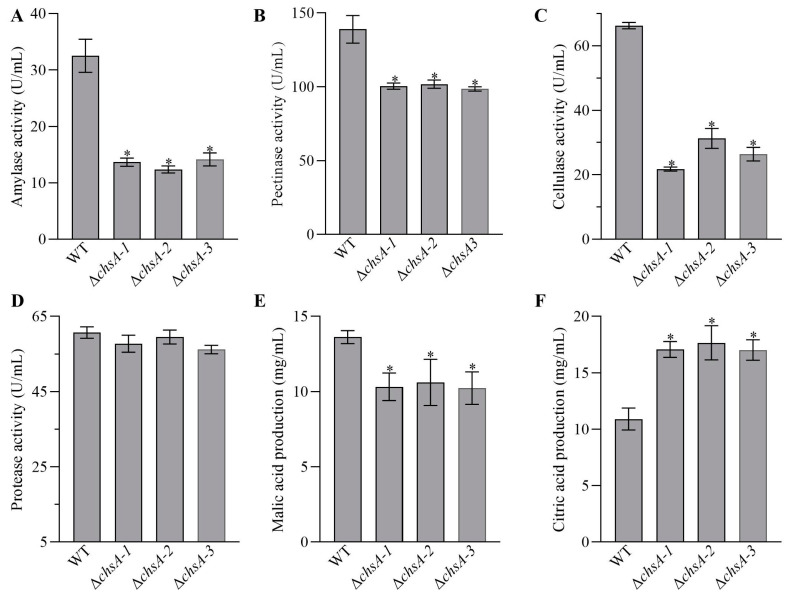
Effects of the deletion of *chsA* on the production of amylases (**A**), pectinases (**B**), cellulases (**C**), proteases (**D**), malic acid (**E**), and citric acid (**F**) when the suspension of conidia from the WT and Δ*chsA* were incubated at WBS with shaking at 30 °C for three days. Asterisked bars in each bar group differ significantly from those unmarked (Tukey’s HSD, *p* < 0.05). Error bars: SDs from three replicates.

The deletion of *chsA* affected the production of amylases, pectinases, cellulases, citric acid, and malic acid rather than the production of proteases. Compared with those in the WT, the activities of amylases, pectinases, and cellulases were lowered by 56.48–61.95%, 26.72–29.06%, and 52.85–60.19%, respectively, in the disruption mutants of *chsA*, but Δ*chsA* exhibited similar protease activity to the WT (Figure 6A–D). Moreover, Δ*chsA* displayed a 22.11–24.88% decrease in the production of malic acid but a 56.09–62.00% increase in the production of citric acid (Figure 6E,F).

## 4. Discussion

In fungi, chitin synthases play important but differential roles in cell wall integrity, cellular growth and development, environmental adaptation, metabolism, and pathogenicity [1,6,7,10,11,14,29,30,31]. In this study, *A. niger* ChsA, a class II chitin synthase, was proven to be involved in conidiation, mycelial morphology, cell wall integrity, cellular response to oxidative stress, and the production of enzymes and organic acids, as discussed below.

Firstly, ChsA is required for asexual conidiation, which is an important stage of the reproductive cycle, and for infection, in mold in nature [24], including in *A. niger*. The deletion of *chsA* in *A. niger* caused severe defects in aerial conidiation on PDA, SDAY, and CZA plates, which was in good agreement with those found in the disruption mutants of *chsA* (a class I CHS-encoding gene), *chsB* (a class II CHS-encoding gene), and *chsC* (a class III CHS-encoding gene) in *A. fumigatus* [7]; *chsI*-*VII* in *M. acridum* [6]; and *chsA* (a class II CHS-encoding gene) in *A. nidulans* [8] and the knockdown mutants of *chs4* (a class III CHS-encoding gene) in *P. chrysogenum* [11]. No significant differences were observed in the diameters of hyphal colonies among the WT and deletion mutants*,* which was similar to the findings in the disruption mutants of *chsA*, *chsB*, and *chsC* in *A. fumigatus* [7], and *chsI*, *chsII*, *chsIV*, and *chsVI* in *M. acridum* [6]*,* but different from the findings in the disruption mutants of *chsIII*, *chsV*, and *chsVII* in *M. acridum* [6]; *chsB* (a class III CHS-encoding gene) in *A. nidulans* [8]; and the knockdown mutants of *chs4* (a class III CHS-encoding gene) in *P. chrysogenum* [11]. Accompanied by the reduction in the yields of conidia, much sparser and smaller conidiophores were observed in Δ*chsA*. Intriguingly, the transcriptional expression of upstream regulators (*fluG*, *flbA*, *flbC*, and *flbE*), velvet regulators (*velB* and *vosA*), and central regulators (*brlA*), which positively controlled conidiation in *Aspergillus* species [32], were significantly decreased in the Δ*chsA* culture grown on PDA plates in *A. niger*. These findings implied that *chsA* was positively involved in conidiation by regulating the transcriptional expressions of some important regulators that are crucial for conidiation in *A. niger*.

Moreover, chitin synthases are important for chitin synthesis. The deletion of *chsA* led to the decrease in chitin content of hyphal cells in *A. niger*, which was in good agreement with the findings in Δ*chsIV*, as well as in Δ*chsVI* in *M. acridum* [6], Δ*chs8* in *F. graminearum* [9], Δ*chsC* (a class Ⅲ CHS-encoding gene) and Δ*chsG* (a class Ⅲ CHS-encoding gene) in *A. fumigatus* [7], and the silencing mutant of *chsC* (a class Ⅲ CHS-encoding gene) in *A. niger* [14]; however, it was opposite to the findings in Δ*chsIV*, Δ*chsVI* in *M. acridum* [6], Δ*chsVII* in *P. graminearum* [10], and Δ*chsD* (a class Ⅶ CHS-encoding gene) in *A. fumigatus* [7]*. It was alo*different from the lack of significant changes in Δ*chsI*, Δ*chsII*, and Δ*chsIII* in *M. acridum* [6], Δ*chsA* (a class Ⅰ CHS-encoding gene), Δ*chsB* (a class Ⅱ CHS-encoding gene), Δ*csmA* (a class Ⅴ CHS-encoding gene), Δ*csmB* (a class Ⅴ CHS-encoding gene), and Δ*chsF* (a class IV CHS-encoding gene) in *A. fumigatus* [7]. Chitin is a vital substance that constitutes the framework of the cell wall and maintains cell wall integrity and structure in fungi [33,34]. Accompanied with the decrease in chitin content, the cell wall of the Δ*chsA* mutants became weakened and fragile, which was evidenced by more protoplasts being released in the Δ*chsA* mutants than in the WT. Moreover, Δ*chsA* mutants exhibited a thicker cell wall than the WT, which was similar to the thickened cell wall in the double deletion mutants of *chsA* (a class II CHS-encoding gene) and *chsC* (a class I CHS-encoding gene) in *A. oryzae* [8], but was contrary to the findings in Δ*chsII*, Δ*chsIII*, Δ*chsIV*, Δ*chsV*, and Δ*chsV* in *M. acridum* [6]. Additionally, Δ*chsA* demonstrated that impaired cell wall integrity led to higher sensitivities to Congo red and SDS in *A. niger*, except for the altered cell wall fragility. The increased sensitivities to Congo red and SDS coincided with those in Δ*chsV* and Δ*chsVI* in *M. acridum* [6], Δ*chs8* in *F. graminearum* [9], Δ*chsVII* in *P. graminearum* [10], Δ*chsB* (a class Ⅱ CHS-encoding gene) in *A. fumigatus* [7], and Δ*csmB* (a class Ⅴ CHS-encoding gene) and Δ*csmA* (a class Ⅴ CHS-encoding gene) in *A. fumigatus* [7], but was in contrast to the increased resistance to Congo red in Δ*chsA* (a class Ⅰ CHS-encoding gene), Δ*chsC* (a class Ⅲ CHS-encoding gene) and Δ*chsG* (a class Ⅲ CHS-encoding gene) in *A. fumigatus* [7], and Δ*chsD* (a class Ⅶ CHS-encoding gene) and Δ*chsF* (a class Ⅳ CHS-encoding gene) in *A. fumigatus* [7].

Apart from the defects in conidiation and cell wall integrity, the deletion of *chsA* led to the formation of looser and smaller pellets and a decrease in the number of septa in hyphal cells. The formation of looser and smaller pellets was opposite to the observation of more compact and/or bigger pellets in the silencing mutants of *chsC* (a class III CHS-encoding gene) in *A. niger* and *chs4* (a class III CHS-encoding gene) in *P. chrysogenum* [11,14]. The fewer septa in the hyphal cells differed from the lack of significant difference among the WT, Δ*chsA* (a class Ⅱ CHS-encoding gene), and Δ*chsC* (a class Ⅰ CHS-encoding gene) in *A. nidulans*, and among the WT, Δ*chs1* (a class Ⅲ CHS-encoding gene), and complementation mutants in *Fusarium asiaticum* [35,36]. Generally, the spores of *A. niger* aggregate fast, subsequently germinate, and then form pellets during submerged fermentation [37]. The deletion of *chsA* inhibited the aggregation of fungal conidia, which might be an explanation for the formation of looser and smaller pellets induced by the absence of *chsA*. In addition, changes in mycelial morphology are often associated with the production of metabolites during submerged fermentation. For example, dispersed mycelium is preferred for the production of gluconic acid and enzymes such as amylases, pectinases, fructofuranosidase, and proteases, whereas the pellet form is desirable for the production of citric acid and oxalate in *A. niger* [14,38,39,40,41,42]. Accompanied by the formation of more compact pellets, the silencing of *chsC* expression in *A. niger* improved the accumulation of citric acid during submerged fermentation [14]. In this study, accompanied by the formation of looser and smaller pellets in Δ*chsA*, the deletion of *chsA* resulted in the decreased production of amylases, pectinases, cellulases, and malic acid, but increased citric acid production. The deletion of *chsA* would simultaneously destroy conidiation and cell wall integrity, which deteriorates the fermentation morphology and reduces the fermentation efficiency [43]. For the decrease in the production of amylases, pectinases, cellulases, and malic acid, the negative effects of metabolic interference outweighed the positive effects of morphological optimization. In addition, both the transport of sugars and ammonia into the cell and the export of citrate ions from the cell are crucial to an understanding of the overproduction of citric acid [37]. The cell wall plays an important role in material transport, so it was speculated that the increases in the fragility of the cell wall and the defects in cell wall integrity caused by the deletion of *chsA* might affect the transport of sugars, ammonia, citrate ions, and so on, which might lead to the increases in citric acid production in Δ*chsA*.

Furthermore, the cell wall is the first line of defense that protects microbes from environmental insults. Therefore, accompanied by the impaired cell wall in *A. niger*, cell tolerances to two typical oxidants (menadione and H_2_O_2_) were decreased in Δ*chsA.* The decreased tolerances to menadione and/or H_2_O_2_ were in accordance with the findings for Δ*chsV* and Δ*chsVII* in *M. acridum* [6], and Δ*chsVII* in *P. graminearum* [10], but differed from the lack of significant changes in Δ*chsI*, Δ*chsII*, Δ*chsIII*, Δ*chsIV*, and Δ*chsVI* in *M. acridum* [6], and Δ*chs8 in F. graminearum* [9]. The CATs and SODs are the two key defense enzymes in the response to oxidative stress; therefore, their total activities are crucial to the response of *A. niger* to menadione and H_2_O_2_ [31,44]. The lowered activities of SODs and CATs coincided with the decreased tolerances caused by the deletion of *chsA* in *A. niger*. However, Δ*chsA* displayed a comparable sensitivity to NaCl and KCl as to the WT, which was similar to that in Δ*chsI*, Δ*chsII*, Δ*chsIII*, Δ*chsIV*, and Δ*chsVI* in *M. acridum* [6], and Δ*chs8* in *F. graminearum* [9], but differed from the increased sensitivity to NaCl observed in Δ*chs1* of *Fusarium asiaticum* [36].

Lastly, the deletion of *chsA* played no roles in the pathogenicity of *A. niger* to the apple, which was similar to in the results observed for Δ*chsI*, Δ*chsII*, Δ*chsIV*, and Δ*chsVI* in *M. acridum* [6], and Δ*chsI*, Δ*chsII*, Δ*chsIII*, Δ*chsIV*, and Δ*chsVI* in *M. acridum* [1], but differed from the reduced virulence in Δ*chsIII*, Δ*chsV*, and Δ*chsVII* in *M. acridum* [6], Δ*chsV* and Δ*chsVII* in *M. acridum* [1], Δ*chs8* in *F. graminearum* [9], and Δ*chsVII* in *P. graminearum* [10].

In conclusion, *chsA* is required for mycelial morphology and cell wall integrity, including the chitin content, adaptive responses to cell wall-disturbing stresses, and the fragility and ultra-structure of the cell wall, and positively affects asexual conidiation, cellular responses to oxidative stresses, and the production of amylases, pectinases, cellulases, and malic acid, but negatively regulates the production of citric acid.

## Data Availability

All data supporting the findings of this study are available from the corresponding author upon reasonable request.

## Supplementary Materials

The following supporting information can be downloaded at: https://www.mdpi.com/article/10.3390/jof9080801/s1; Figure S1: Phylogenetic analysis, diagram and identification for the disruption of *chsA* in *A. niger*; Figure S2 Images of formed conidiophores during incubation for 3 days on PDA, SDAY, and CZA at 30 °C; Table S1: The primers used for *chsA* gene manipulation. Table S2. The primers used for qRT-PCR.
