# Supplementary material for: ChsA, a Class Ⅱ Chitin Synthase, Contributes to Asexual Conidiation, Mycelial Morphology, Cell Wall Integrity, and the Production of Enzymes and Organic Acids in Aspergillus niger
Source: J Fungi (Basel). 2023 Jul 29;9(8):801. doi: 10.3390/jof9080801 (PMC10455844; doi:10.3390/jof9080801)
Supplement: Supplementary file 1 [file jof-09-00801-s001.zip › jof-2509220-supplementary.pdf]

Table S1. The primers used for *chsA* gene manipulation

Primers	Paired sequences (5′–3′)*	Purpose
hygB-F/R	aaaaaGAGCTCGACGTTAACTGATATTGAAGGAGC/ aaaaaCTCGAGAACCCAGGGGCTGGTGAC	PCR the expression cassette of *hygB*
chsA-F/R	ATGATATACGAAATGATGGTTATGAAGCG/ CTATCTCACCGCCAGCACGG	Cloning ORF of *chsA*
5′*chsA*-F/R	aaaaaCCCGGGAAGGTGGTATGGGCTGGACT/ aaaaaCCCGGGACGCAAAAAAGCGGAGACAG	Cloning *chsA* 5′ (1564bp)
3′*chsA*-F/R	aaaaaCTCGAGACGAGGATGAGATTGGCTTC/ aaaaaTCTAGAACTACCACGGACCACAGGAT	Cloning *chsA* 3′ (2059bp)
p*chsA*-F/R	CCTTGTTTGGTATCTGCCCT/ CAATCTTCTTCCAACCATCC	PCR detecting *chsA*
q*chsA*-F/R	CCCGGGAAGGTGGTATGGGCTGGACT/ CCCGGGACGCAAAAAAGCGGAGACAG	qPCR detecting *chsA* gene
q*β-tubulin*-F/R	TCAAGATGTCCTCTACCT/ GGAACATAGCAGTGAACT	qPCR detecting tubulin gene

* Underlined regions denote the sites of restriction enzyme for disruption of *chsA* (*Xma*I/*Sma*I and *Xho*I/*Xba*I) through homogenous recombination of 5′ and 3′ fragments separated by hygB marker and for the expression cassette including the promoter and open reading frame of hygB (*Sac*I/*Xho*I).

Table S2. The primers used for qRT-PCR

Tag code	Gene	Annotation	Sequences (5'-3') of paired primers
gene08183	FluG	Upstream development activator	AAGATTGCTGCTGCTGATA/GTCGTAGGTAATCGTCTCATT
gene03944	FlbA	Upstream development activator	AGCAGCATCTCTACTTCAG/CTCCTTCGCCATCTTCAC
gene04120	FlbC	Upstream development activator	CTACTATTCCGCCTCTGT/GCCATTCTCGTAAGATTCC
gene06270	FlbB	Upstream development activator	CTCTTCCTTCCATCTCTA/TCATTCTAGTACGCTTTG
gene04684	FlbD	Upstream development activator	TGGACATGAGACAGCAATAC/AGAGATCGTGGTAGGCATA
gene02514	FlbE	Upstream development activator	TGGTGGATTGTGTATAAC/CTCATCATACCCATCATC
gene00348	LaeA	Developmental repressor	GGACTTGGAGAGAATCAG/AGATGTTGTAAGCGTGTAT
gene02682	VeA	Developmental activator	GTTCCTCTTACACTTCTCAT/GAGTTCCAGGTAGTTGTG
gene00832	SfgA	Developmental repressor	CGAACAACCTGCTTATAGTC/GCTGCTTGAATCTCCATC
gene04565	VelB	Developmental repressor; Regulation for conidial maturation	CCTCAGATGAACAATTAC/TAGTAAGGCTGATAATAGG
gene04761	AbaA	Regulation for sterigmata formation	AACATCCTTTCCTTACCT/GTCATCCATCAACTTCAG
gene04357	WetA	Regulation for conidial maturation	CAGTGTGGATGGCAACAA/CGCTGGACAATGACTCAAG
gene10505	VosA	Regulation for conidial maturation	ATGTGGATAATACTGATG/ATCTATCTGTGATGATTG
gene00550	BrlA	Initiator for conidiogenesis	AGAACCTTCCGTCTATTATCA/ATGCTCTTGCCTCTTGAA
gene05820	StuA	Modulator for conidiophore formation	ATATGATTAACGGCACAA/CTCACTCTTCAGAATACC
gene02843	*β*-tubulin	internal standard	TCAAGATGTCCTCTACCT/GGAACATAGCAGTGAACT

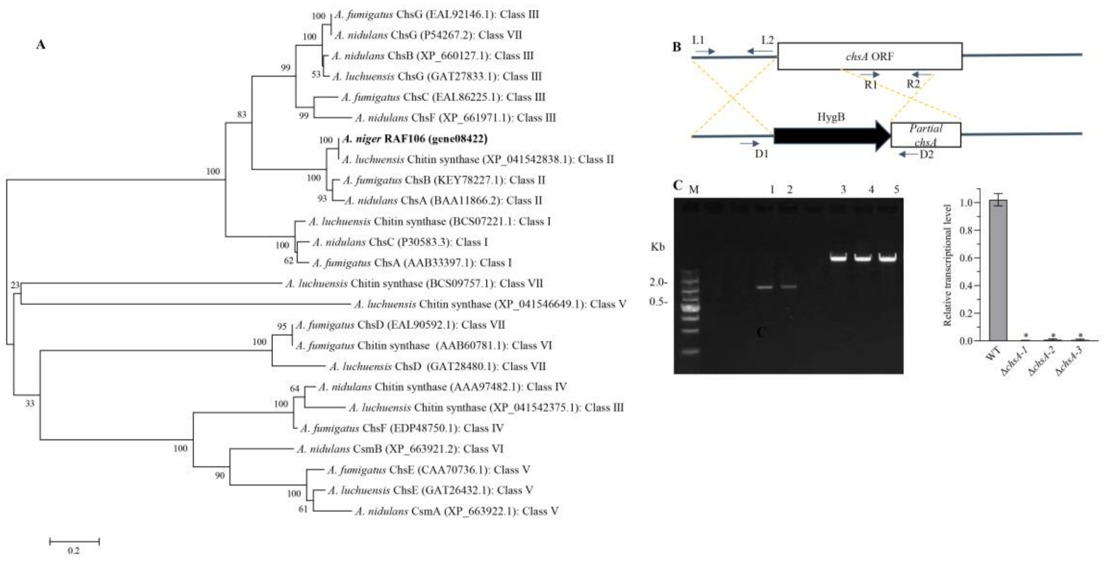

Figure S1 Phylogenetic analysis, diagram and identification for the disruption of *chsA* in *A. niger*. (A) Phylogenetic tree constructed for *chsA* in *A. niger* and other 24 CHSs belonging to 7 classes (classes I to VII) from *A. fumigatus*, *A. nidulans*, and *A. luchuensis* in the NCBI database (accession codes given in parentheses) using a neighbor-joining method. Scale bar: branch length proportional to genetic distance. (B) Diagram for the disruption of *chsA*. L1/L2 and L3/L4: paired primers used for cloning the 5′ and 3′ regions of *chsA*, respectively. D1/D2: paired primers used for PCR detection of disrupted target gene. (C) Identifying the disruption of *chsA* by PCR (left) and qRT-PCR (right). M: maker, Lane 1, 2, 3, 4, and 5: WT, WT, disruption mutant 1, disruption mutant 2, and disruption mutant 3, respectively.

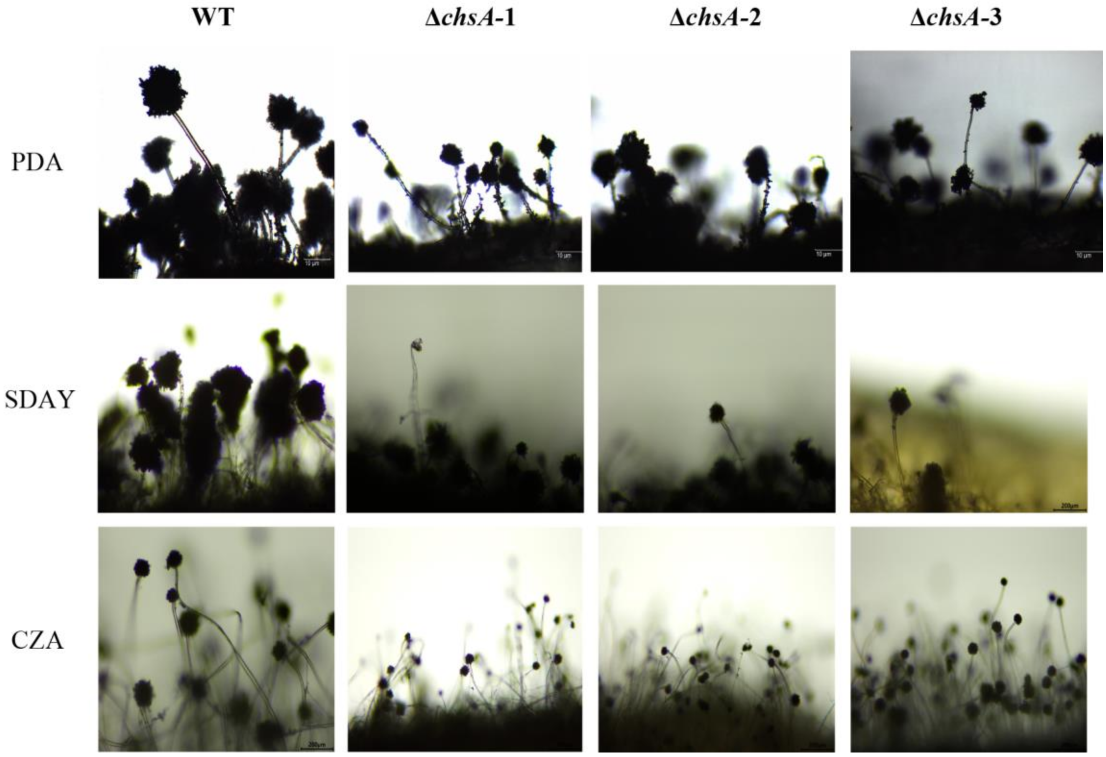

Figure S2 Images of formed conidiophores during incubation for 3 days on PDA, SDAY, and CZA at 30 °C.
